# ABA signalling and metabolism are not essential for dark-induced stomatal closure but affect response speed

**DOI:** 10.1038/s41598-021-84911-5

**Published:** 2021-03-11

**Authors:** Ashley J. Pridgeon, Alistair M. Hetherington

**Affiliations:** School of Biological Sciences, Life Sciences Building, University of Bristol, 24 Tyndall Avenue, Bristol, BS8 1TQ UK

**Keywords:** Abiotic, Light stress, Plant cell biology, Plant physiology, Plant signalling

## Abstract

Stomata are microscopic pores that open and close, acting to balance CO_2_ uptake with water loss. Stomata close in response to various signals including the drought hormone abscisic acid (ABA), microbe-associated-molecular-patterns, high CO_2_ levels, and darkness. The signalling pathways underlying ABA-induced stomatal closure are well known, however, the mechanism for dark-induced stomatal closure is less clear. ABA signalling has been suggested to play a role in dark-induced stomatal closure, but it is unclear how this occurs. Here we investigate the role of ABA in promoting dark-induced stomatal closure. Tracking stomatal movements on the surface of leaf discs we find, although steady state stomatal apertures are affected by mutations in ABA signalling and metabolism genes, all mutants investigated close in response to darkness. However, we observed a delayed response to darkness for certain ABA signalling and metabolism mutants. Investigating this further in the quadruple ABA receptor mutant (*pyr1pyl1pyl2pyl4*), compared with wild-type, we found reduced stomatal conductance kinetics. Although our results suggest a non-essential role for ABA in dark-induced stomatal closure, we show that ABA modulates the speed of the dark-induced closure response. These results highlight the role of ABA signalling and metabolic pathways as potential targets for enhancing stomatal movement kinetics.

## Introduction

Plants are able to rapidly respond to environmental stimuli by regulating gas exchange through changes in the aperture of stomata on leaf surfaces. Stomata are composed of two guard cells surrounding a central pore. Changes in guard cell turgor pressure bring about changes in the aperture of the stomatal pore. The regulation of gas exchange allows plants to balance the uptake of carbon dioxide (CO_2_) with the loss of water^1^. Much work has focused on the underlying mechanisms of stomatal movements, especially on how ion transport across guard cell membranes helps to mediate changes in guard cell turgor pressure, the mechanisms behind blue and red light induced stomatal opening, and how the drought hormone abscisic acid (ABA) leads to stomatal closure and the inhibition of stomatal opening^2^. However, the process underlying dark-induced stomatal closure is unclear. It is unknown whether darkness actively accesses stomatal closure machinery or whether it is a passive response to the absence of light. In addition, as there is an increase in leaf intercellular CO_2_ concentration (Ci) during dark treatment it is possible that CO_2_ also has a contribution to make towards dark-induced closure. However, the contribution of Ci to dark-induced closure has not been investigated in the present study.

Few mutants have been identified that show strongly defective dark-induced stomatal closure responses. Those that do are often involved in regulating stomatal opening; such as mutants in COP1, an E3 ubiquitin ligase that functions downstream of the cryptochrome and phototropin photoreceptors^3^, or the dominant mutation, *ost2-2D,* causing constitutive activation of the H^+^ ATPase AHA1 (a proton pump that hyperpolarises guard cell membranes, inducing ion transport and ultimately stomatal opening)^4^. Additionally, reorganisation of the actin cytoskeleton has been shown to be crucial for stomatal dark-induced closure, as mutations within the Arp2/3 and SCAR/WAVE complexes that control actin cytoskeleton dynamics show lack of dark-induced closure^5,6^. Weaker phenotypes have also been observed, such as in the *myb61* transcription factor mutant, where stomatal apertures are increased in dark conditions, however here, stomatal apertures are also increased in light conditions and there is a noticeable but reduced response to darkness^7^. More recently additional signalling components required for dark-induced closure have been identified. The pseudokinase GHR1, involved in activating the downstream ion channel SLAC1, has been shown to be required for stomatal closure in response to a number of signals, including darkness^8^. A MEK1/MPK6 signalling cascade activated by H_2_O_2_ (produced by RBOHD and RBOHF) and culminating in the production of NO (by NIA1) has also been shown to be required for dark-induced closure^9^.

Studies have linked ABA signalling to dark-induced closure, but the precise way in which it is involved in regulating dark-induced closure is unclear. Microarray data have shown components of the ABA signalling pathway undergo transcriptional regulation in response to darkness, however these changes are likely to reflect longer term adaptation rather than the short term closure^10^. Additionally, another study has shown that a selection of ABA receptor mutants (*pyr1pyl1pyl2pyl4*,* pyr1pyl4pyl5pyl8, pyr1pyl2pyl4pyl5pyl8*, *pyr1pyl1pyl2pyl4pyl5pyl8*) all show increased stomatal conductance under light and dark conditions. However, when comparing the change in stomatal conductance from light to dark conditions all of the previously mentioned ABA receptor mutants (except the strongest mutant, *pyr1pyl1pyl2pyl4pyl5pyl8*) show changes in stomatal conductance similar to wild type. Similarly, mutations within PP2C phosphatases (downstream negative regulators of ABA signalling) and ABA degradation mutants, affect stomatal conductance without preventing responses to darkness. In the ABA biosynthesis mutants, *aba1-1* and *aba3-1,* stomatal conductance is also increased, however both mutants still respond to a dark (although this appears weakened in *aba3-1*)^11^. In addition, stomatal aperture changes in the PP2C mutants *abi1* and *abi2* show reduced responses to darkness^12^. This suggests a situation where ABA signalling may make a contribution to dark-induced stomatal closure, however, it also suggests that ABA has more general effects on stomatal apertures regardless of light or dark conditions.

Here we investigate how defects in ABA signalling and metabolism affect stomatal response to darkness and light. We analyse the movement of stomata through direct measurements on leaf discs and through monitoring changes in stomatal conductance. We find evidence that ABA signalling is not essential for dark-induced closure. However, we provide additional evidence that defects in ABA signalling and metabolism affect the timing of stomatal responses to both light and darkness. Overall, we conclude that ABA signalling does not play a major role in mediating dark-induced closure but does play a role in modulating the speed of closure.

## Results

### ABA signalling and biosynthesis mutant responses to darkness

To explore whether mutations in ABA biosynthesis, degradation or signalling genes affect stomatal responses to darkness, stomatal movements were measured in leaf discs from ABA metabolism and signalling mutants. Aperture measurements taken over a 2 h dark treatment time course, with independent leaf disc measurements at each timepoint, allowed for detection of trends in stomatal responses. Of the 14 member ABA receptor family, quadruple and sextuple ABA receptor mutants (*pyr1pyl1pyl2pyl4*^13^—*q1124* and *pyr1pyl1pyl2pyl4pyl5pyl8* ^14^—*s112458*) were used. The ABA biosynthesis double mutant *nced3nced5*^15^ (*nced3/5*—a double mutant in the *NCED3* and *5* genes which catalyse the first committal step in ABA biosynthesis, thought to be the rate limiting step under drought conditions^15–17^) and mutants within 2 genes involved in rapid ABA activation from inactive glucose esters (*bg1* and *bg2*^18^) were used. Additionally mutants in ABA hydroxylation genes (*cyp707a1* and *cyp707a3*^19^) involved in ABA catabolism were used (exact mutant accession codes are shown in the methods section).

**Figure 1:**
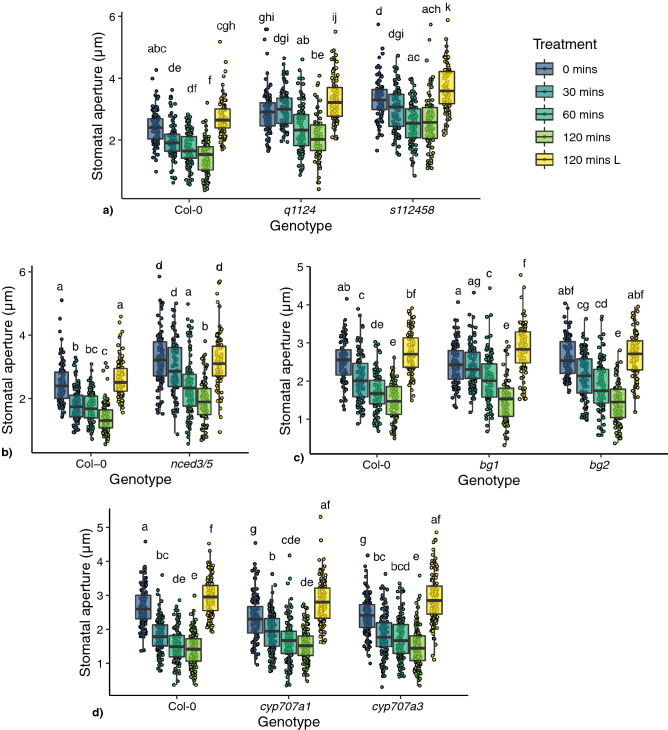
Stomatal responses of ABA signalling, biosynthesis and degradation mutants to darkness. Stomatal responses to darkness over a 120 min time course were tracked in leaf discs harvested from (**a**) ABA receptor mutants (*q1124* and *s112458*), (**b**) *nced3/5*, (**c**) ABA activation mutants (*bg1* and *bg2*) and (**d**) ABA degradation mutants (*cyp707a1* and *cyp707a3*). Leaf discs were incubated under light for 120 mins before transfer to darkness. 120 mins L represent aperture data from leaf discs left under light for 120 mins. n = 90 from 9 individual plants over 3 independent experiments. Data is presented in boxplots showing the median and interquartile range of each group. The upper and lower whiskers represent data within 1.5 * the interquartile range. All data values are represented by points. Data statistically analysed using 2-way ANOVA with Tukey multiple comparison tests, letters denote significant differences at p < 0.05.

Delays in stomatal closure following dark treatment were observed for the ABA receptor mutants *q1124* and *s112458*, the ABA biosynthesis mutant *nced3/5*, and the ABA activation mutant *bg1,* which all show no significant change in stomatal aperture, compared with wild type, after 30 mins dark treatment (Fig. 1a–c). No delays in dark-induced stomatal closure were observed in the ABA activation mutant *bg2* or the ABA catabolism mutants *cyp707a1* or *cyp707a3* (Fig. 1c,d). Absolute changes in stomatal aperture for each mutant are shown in Fig. S1. The absence of a delay phenotype in the *bg2* mutant could be due to the difference in subcellular location and/or reduced activity of the BG2 protein compared with BG1^18^. Additionally, mutations in *CYP707A1 and 3* genes lead to increased levels of ABA^19,20^ and increased ABA signalling activity (the opposite of what occurs in ABA biosynthesis and signalling mutants), explaining the lower starting apertures (as observed previously^19^) and potentially the absence of delay.

In addition to delays in dark-induced stomatal closure, changes to stomatal apertures regardless of treatment are observed for many of the mutants analysed. The ABA receptor and biosynthesis mutants (*q1124*, *s112458* and *nced3/5*) show significantly increased stomatal apertures before (*q1124*—p = 0.00003, *s112458*—p < 0.00001, *nced3/5—*p < 0.00001) and after 2 h of dark treatment compared with Col-0 (*q1124—*p < 0.00001, *s112458—p* < 0.00001, *nced3/5*—p < 0.0001). Conversely, the ABA catabolism mutants (*cyp707a1* and *cyp707a3*) show reduced stomatal apertures prior to dark treatment (*cyp707a1—*p = 0.00268, *cyp707a3*—p = 0.02927) and similar apertures after 2 h dark treatment. The ABA biosynthesis mutants *bg1* and *bg2* show no difference when compared with Col-0 before or 2 h after dark treatment.

### Stomatal conductance responses to darkness

**Figure 2:**
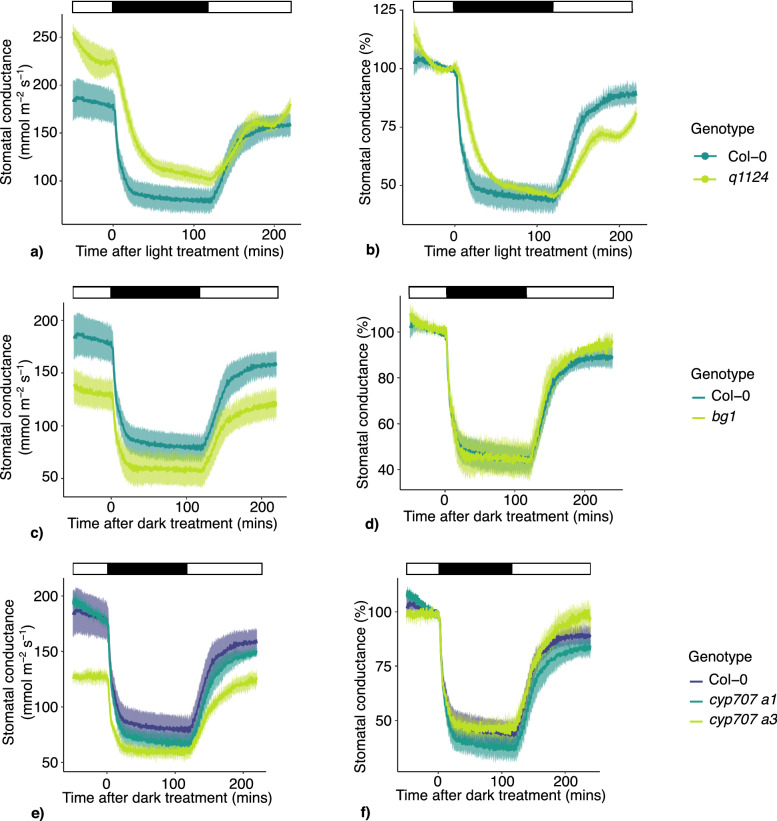
Stomatal conductance responses to darkness for ABA signalling, activation, and degradation mutants. Stomatal conductance of ABA signalling and metabolism mutants were measured in response to 120 mins darkness, followed by a further 120 mins light. Absolute and relative stomatal conductance values for (**a**, **b**) *q1124*, (**c**, **d**) *bg1* and (**e**, **f**) *cyp707a1* and *cyp707a3* are shown. Lines represent the mean ± s.e.m. 3–4 plants were measured per genotype. Light treatment is represented above each plot, with black boxes representing darkness and white boxes representing light.

**Figure 3:**
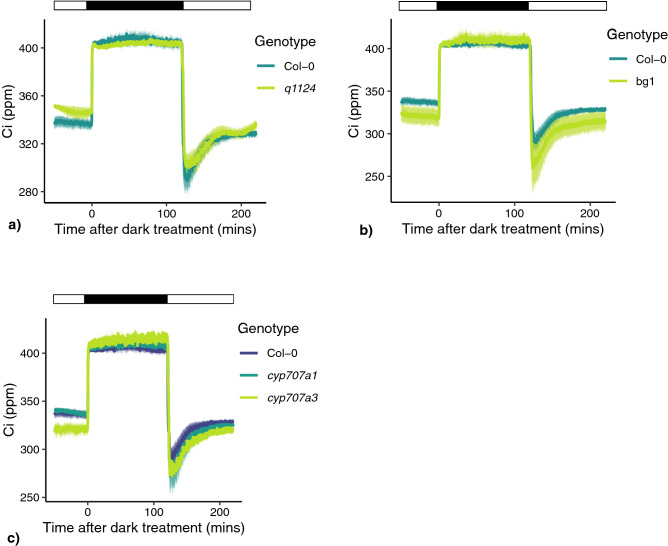
Intercellular CO_2_ concentration in response to darkness applied at midday. Intercellular CO_2_ concentration (C_i_) of leaves of (**a**) ABA receptor mutant (*q1124*), (**b**) ABA activation mutant (*bg1*), (**c**) ABA degradation mutants (*cyp707a1* and *cyp707a3*) in response to darkness applied at midday. Lines represent the mean ± s.e.m. 3–4 plants were measured per genotype. Light treatment is represented above each plot, with black boxes representing darkness and white boxes representing light.

**Figure 4:**
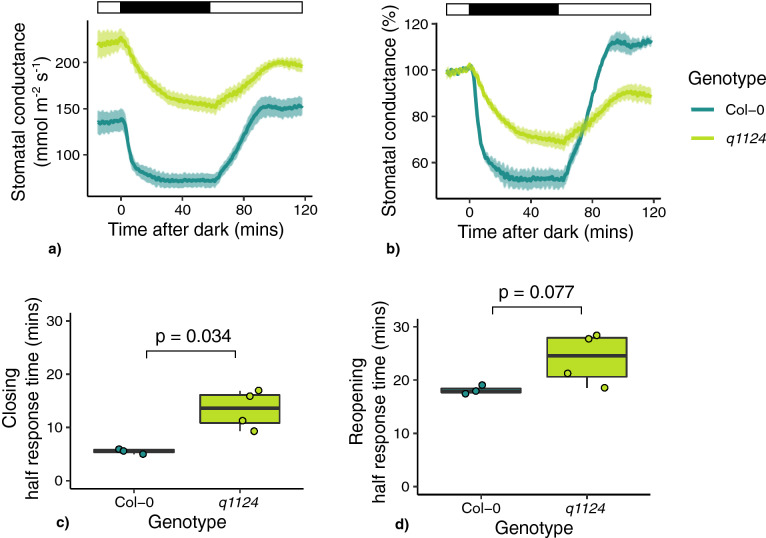
*q1124* stomatal conductance responses to darkness at midday. Stomatal conductance of *q1124* ABA quadruple receptor mutant was measured in response to darkness applied 4–5 h post dawn. (**a**) Absolute and (**b**) relative stomatal conductane values are presented as the mean ± s.e.m. The time taken to reach half of the maximum stomatal conductance change (half response times) in response to (**c**) darkness and (**d**) light are presented in boxplots showing the median and interquartile ranges. 3–4 plants measured per genotype. Light treatment is represented above each stomatal conductance plot, with black boxes representing darkness and white boxes representing light.

**Figure 5:**
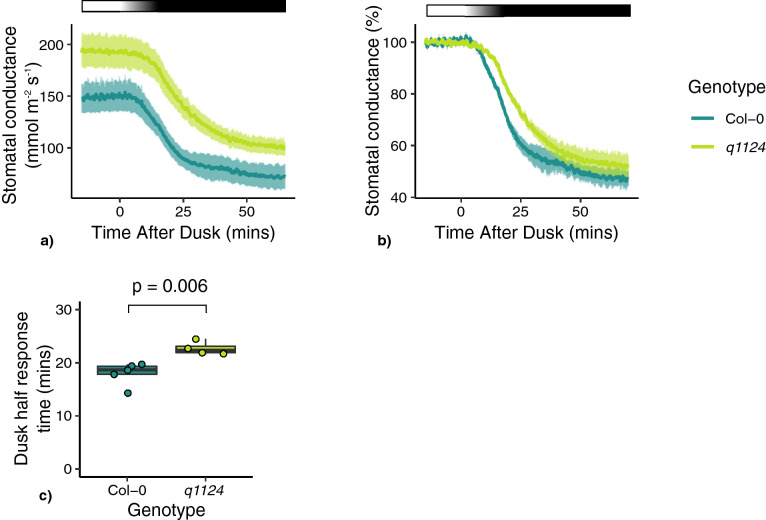
*q1124* stomatal conductance response at dusk. Stomatal conductance of the *q1124* quadruple ABA receptor over the dusk transition period. On the onset of dusk, the lights were dimmed over a 15 min period until completely turned off. (**a**) Absolute and (**b**) relative stomatal conductance values are presented as the mean ± s.e.m. (**c**) The time taken to reach half of the maximum stomatal conductance change (half response times) upon dusk are presented in boxplots showing the median and interquartile ranges. 4–5 plants measured per genotype. Light treatment is represented above each stomatal conductance plot, with black boxes representing darkness and white boxes representing light. A gradient represents the 15 min transition from light to darkness.

**Figure 6:**
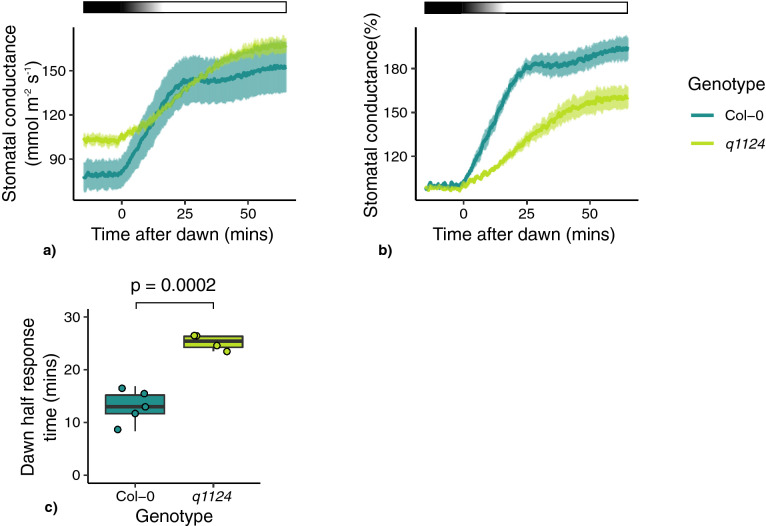
*q1124* stomatal conductance responses at dawn. Stomatal conductance of the *q1124* quadruple ABA receptor over the dawn transition period. On the onset of dawn, the lights were turned on over a 15 min period until reaching full brightness. (**a**) Absolute and (**b**) relative stomatal conductance values are presented as the mean ± s.e.m. (**c**) The time taken to reach half of the maximum stomatal conductance change (half response times) upon dusk are presented in boxplots showing the median and interquartile ranges. 4–5 plants measured per genotype. Light treatment is represented above each plot, with black boxes representing darkness and white boxes representing light. A gradient represents the 15 min transition from darkness to light.

**Figure 7:**
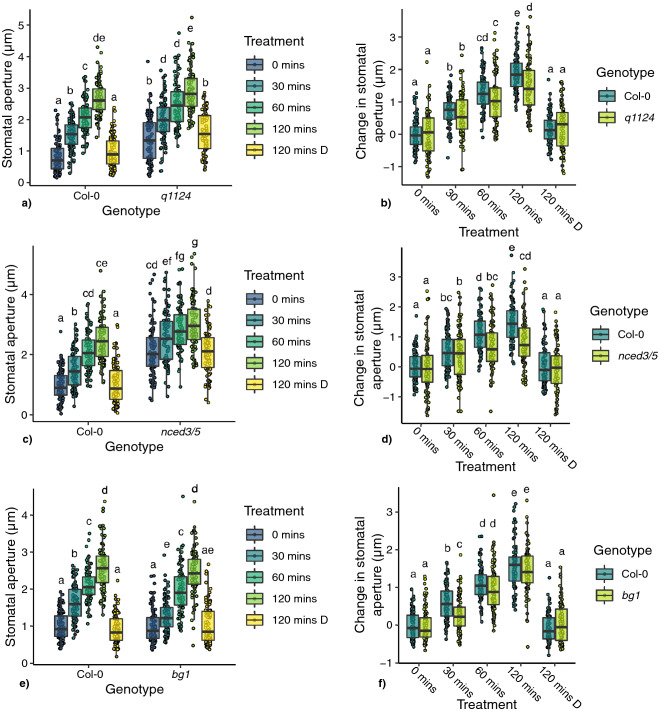
Stomatal responses of ABA biosynthesis and signalling mutants to light. (**a**, **c**, **e**) show absolute stomatal apertures and (**b**, **d**, **f**) show absolute change in stomatal aperture for *q1124*, *nced3/5*, and *bg1* respectively. Leaf discs were harvested under green light before dawn and incubated in darkness for 2 h before treatment. Treatment times refer to time in light (other than 120 mins D—which refers to 120 mins in darkness). Data is presented in boxplots showing the median and interquartile range of each group. The upper and lower whiskers represent data within 1.5 × the interquartile range. All data values are represented by points. Data was analysed using a 2-way ANOVA with Tukey HSD multiple comparison test, letters denote significant differences at p < 0.05.

The stomatal conductance responses to darkness applied at midday were also measured in the following genotypes; Col-0, *q1124*, *bg1*, *cyp707a1*, and *cyp707a3*. Due to the greatly reduced leaf size phenotype of the *s112458* and *nced3/5* mutants, stomatal conductance could not be recorded. Focusing on dark-induced decreases in stomatal conductance, it is evident this response occurs much faster than dark-induced closure in leaf discs (Fig. 1). The absolute and relative stomatal conductance values are shown for the mutants in Fig. 2a, c, e and b, d, f respectively. Here, delayed responses are only noticeable for the *q1124* receptor mutant (Fig. 2a,b). All mutants appear to decrease their stomatal conductance by around 50% in response to darkness. The difference in the periods of delays observed in leaf discs and in measurements of stomatal conductance may reflect that stomatal conductance responses are faster than changes in stomatal aperture measured on leaf discs, similar to observed differences in stomatal movement when comparing responses to red light in epidermal peels and intact leaves^21^. Stomatal conductance responses generally reach their maximum by 25 mins (Figs. 2, 4, 5, 6) whereas leaf disc stomatal aperture responses reach their maximum by 120 mins (Figs. 1, 7). The slower movements on leaf discs may allow for more subtle mutant phenotypes to be identified. Additionally, when tracking the intercellular leaf CO_2_ concentration (C_i_) over the course of the experiments a rapid increase is seen upon dark treatment, that then rapidly decreases upon reintroduction to light, before returning to levels comparable to those before the initial dark treatment (Fig. 3). This build-up of C_i_ may contribute to the stomatal conductance response, however this requires further investigation.

### Further analysis of *q1124* stomatal conductance responses to darkness

For Col-0 and *q1124*, the stomatal conductance responses were measured when whole plants were placed in darkness 4–5 h after dawn. Leaves were clamped into the gas analyser leaf cuvette, 2 h prior to the onset of darkness. After 1 h of darkness plants were reintroduced to light for a further hour. *q1124* shows higher stomatal conductance (at time 0—p = 0.00231) and stomatal conductance is reduced to a lesser extent than wild type in darkness. This is true in absolute (Fig. 4a) and relative (Fig. 4b) terms. The time taken for *q1124* to reach half of its total stomatal conductance response to darkness (darkness half response time—Fig. 4c) and to light (light half response time—Fig. 4d) shows greater variability when compared with Col-0, and the q1124 half response time in response to darkness is significantly increased (p = 0.0148). However, there do appear to be differences in the response of *q1124* to darkness when comparing Figs. 2a, b and 4a, b suggesting a degree of biological variation in the *q1124* dark response phenotype.

Stomatal conductance in Col-0 and *q1124* was also measured at dusk. Unlike the response to darkness measured at midday, where plants were plunged into darkness, the onset of dusk was marked with a 15 min transition from light to dark. The absolute stomatal conductance of Col-0 and *q1124* is shown in Fig. 5a. It is clear that both genotypes respond to dusk. Because of the differences in absolute initial stomatal conductance, relative stomatal conductance were calculated. The data in Fig. 5b suggest that the speed of stomatal conductance change in the *q1124* ABA quadruple receptor mutant is reduced compared with Col-0. The difference between Col-0 and *q1124* is less pronounced than that observed in Fig. 4, yet measurement of dusk half response times supports a slower stomatal conductance response of *q1124* to darkness (p = 0.00607).

### The effect of mutations in ABA biosynthesis, signalling and activation on stomatal responses to light

In addition to the delay in dark-induced stomatal closure and slower stomatal conductance responses observed in *q1124*, the data in Figs. 2 and 4 suggest that there might also be defects in the light-induced opening response of the quadruple receptor mutant. Stomatal conductance of *q1124* was measured over the dawn period. Here, similarly to dusk, the onset of dawn was marked with a 15 min transition period from dark to light. Absolute and relative stomatal conductance values are presented in Fig. 6a, b respectively. Similar to the response observed at dusk, in comparison to wild type, the *q1124* mutant shows increased absolute stomatal conductance values and an increased half stomatal conductance response time, suggesting this response also occurs at a slower rate (Fig. 6c) (p = 0.000239).

The stomatal movements of *q1124*, *nced3/5* and *bg1* mutants were also analysed in leaf discs. Here, leaf discs were harvested pre-dawn under green light, incubated in the dark for 2 h, before being transferred to light. The apertures were monitored over a 2 h time course. Figure 7 shows both the absolute and relative change in stomatal aperture for the three mutants. It is evident that both *q1124* and *nced3/5* have significantly increased stomatal apertures at 0 mins (p < 0.0001, p < 0.0001 respectively), whereas *bg1* is similar to Col-0. This makes comparisons between the absolute stomatal apertures of Col-0, *q1124* and *nced3/5* more difficult to interpret. However, when analysing the absolute change in stomatal aperture for both *q1124* and *nced3/5* there are no initial significant differences at 30 mins, but by 120 mins there are significantly reduced responses (p < 0.005, p < 0.0001 respectively).

Unlike *q1124* and *nced3/5*, the *bg1* mutant shows a response analogous to that observed when plants are placed in darkness (except instead of an initial delay in dark-induced stomatal closure, here a delay in light-induced stomatal opening is observed). *bg1* shows a significantly weakened response at 30 mins (p < 0.0005), before the *bg1* mutant eventually catches up to Col-0 by 60 and 120 mins.

## Discussion

ABA is well known as a regulator of seed dormancy and plant responses to drought including reductions in stomatal aperture and inhibition of light-induced opening^22^. Additionally, evidence is emerging supporting roles for basal ABA signalling under non-stress situations^23^. On a molecular level, a subset of ABA receptor family members (subfamily 1) are known to activate downstream signalling under basal amounts of ABA^24^ and mutation of ABA signalling and metabolism components alters plant growth and development under non stress conditions^25,26^. Here we explore the role of ABA and ABA signalling in stomatal responses to the onset of light and darkness.

The mechanisms behind light-induced stomatal opening are relatively well understood. Stomatal opening in response to light is driven by the activity of plasma membrane H^+^ATPases. This generates a proton gradient across the guard cell plasma membranes, resulting in membrane hyperpolarization leading to the influx of cations and anions, changes in guard cell turgor pressure, and ultimately the opening of stomata. Blue and red light promote stomatal opening via independent pathways. Blue light-induced opening is predominantly initiated via activation of phototropin photoreceptors within the guard cell^27^, whereas red light-induced opening is dependent on photosynthetic electron transport^28,29^. When Arabidopsis plants are moved from light to dark their stomata close, however, the mechanisms behind dark-induced stomatal closure are less clear^30^. Studies have linked ABA signalling to dark-induced closure, but it is unclear to what extent ABA signalling is required for this process^4,10–12^.

Here, we present evidence that supports a non-central role for ABA metabolism and ABA signalling in dark-induced stomatal closure. In leaf discs we observe that all ABA signalling and metabolism mutants analysed were able to respond to darkness (Fig. 1). Compared with wild type, ABA receptor mutants (*q1124* and *s112458*) and the ABA biosynthesis mutant (*nced3/5*) showed increased stomatal apertures before treatment whereas ABA degradation mutants (*cyp707a1* and *cyp707a3*) showed decreased stomatal apertures. This is in line with previous reports, namely that, defects in ABA signalling and production lead to increases in steady state stomatal apertures and transpiration, whereas defects in ABA degradation lead to the opposite^11,14,15,20^. This shows links between ABA, ABA signalling and the regulation of stomatal apertures under non stress conditions. However, following dark treatment *cyp707a1* and *cyp707a3* mutants close to the same extent as wild type. On the other hand, the stomata of *q1124, s112458,* and *nced3/5* mutants all remain more open than wild type. These results are similar to those observed for stomatal conductance in ABA receptor mutants (*q1124*/*pyr1pyl1pyl2pyl4, pyr1pyl4pyl5pyl8, pyr1pyl2pyl4pyl5pyl8*) in Merilo et al. 2013 with one exception. Whereas Merilo et al. 2013 report no response to darkness, here we report that the stomata of *s112458* mutant are still able to respond to darkness, but to a lesser extent than wild type. The strong ABA biosynthesis mutant *nced3/5* also behaves similarly to the *aba1-1* and *aba3-1* biosynthesis mutants in Merilo et al. 2013. *nced3/5* shows significantly more open stomata throughout the experiment and responds to darkness, although to a lesser extent than wild type. Additionally, we report ABA signalling (*q1124* and *s112458*) and biosynthesis mutants (*nced3/5* and *bg1*) show a delay in closure, with no significant change in stomatal aperture following 30 min of dark treatment, when measured in leaf discs. In Merilo et al. 2013, T-DNA insertion mutants in the downstream ABA signalling component OST1 (*ost1-3*) and the ion channel SLAC1 (*slac1-3*) show similar dark-induced transpiration changes to those of ABA biosynthesis mutants (*aba1-1* and *aba3-1*), where stomatal conductances are increased but still show responses to darkness. This suggests a non-central role for ABA and ABA signalling in dark-induced closure.

Exploring this further we find that the time taken for *q1124* mutant to reach its maximum stomatal conductance response is increased compared to Col-0 (Figs. 2, 4, 5, 6). This is observed when darkness and light are applied during the middle of the day and also at the dawn and dusk transition periods. This suggests that although ABA signalling is not essential for dark-induced closure, it appears to be involved in increasing the speed of stomatal responses to darkness and light. It should be noted there is a difference between the time taken for stomatal apertures to show maximum responses (Fig. 1) and stomatal conductance responses to reach their maximum (Figs. 2, 4, 5, 6), with stomatal movement on leaf discs appearing slower than that of changes in conductance. The reasons for this are unclear but likely due to differences between the systems of a leaf disc and an attached leaf.

Manipulating the speed of stomatal responses has been shown to increase biomass accumulation^31^ and may improve key plant processes such as photosynthetic carbon assimilation and water use efficiency^32^, suggesting it could be a key target for plant breeders. How defects within ABA metabolism and signalling are affecting the speed of stomatal responses is currently unclear but may stem from altered amounts or activities of further signalling components and/or ion channels within plant cells. Additionally, exogenous ABA application has been reported to alter stomatal kinetic responses to changes in light conditions in gymnosperms, suggesting roles for ABA in modulating stomatal kinetics across taxa^33^.

At the onset of darkness there is a rapid increase in CO_2_ within the leaf (C_i_), which rapidly decreases upon the re-introduction of light (Fig. 3). As photosynthetic CO_2_ fixation ceases in darkness an increase in C_i_ is not surprising. However, the role of C_i_ in driving stomatal responses is unclear. Some studies suggest that under changing light conditions C_i_ allows interaction between photosynthetic assimilation rate (A) and stomatal conductance, making C_i_ a potential candidate for coordinating mesophyll and stomatal responses to light^34^. However, other studies show that stomatal conductance responses to changes in light still occur when C_i_ is kept constant and in mutants where C_i_ is increased^28,35^. This has led to the suggestion that other signals, not C_i_, are involved in coordinating stomatal responses with photosynthetic activity^1^. Regardless, the increase of C_i_ in response to darkness is highlighted here (Fig. 3) but its contribution to stomatal responses to changes in light is beyond the scope of this study.

Similar to our conclusions regarding the role of ABA in guard cell dark signalling, it has been reported that CO_2_-induced stomatal closure proceeds through an ABA-independent pathway downstream of OST1 and that basal ABA signalling enhances CO_2_-induced closure^36,37^. However, there is disagreement as to the precise role of ABA in stomatal responses to CO_2_, as other studies suggest that ABA and ABA signalling are required for elevated CO_2_ induced stomatal closure^38^. Most recently, a study has shown ABA catabolism plays a role in regulating stomatal responses to changes in CO_2_ concentration both on a physiological and developmental scale^39^.

In conclusion, our results using ABA signalling and metabolism mutants show that stomatal conductance and stomatal apertures decrease in response to darkness. The differences between the mutants and the wild type were reflected in the slower rates of closure and stomatal conductance changes found in the mutants. While our results do not support a primary role for ABA in the events underlying dark-induced stomatal closure, we find a role for this hormone in modulating the speed of reaction. This highlights the role of ABA in regulating stomatal aperture/transpiration under non-stress conditions.

## Methods

### Plant material and growth conditions

*Arabidopsis thaliana* was grown in a 3:1 all purpose compost (Sinclair): silver sand (Melcourt) mixture. Seeds were stratified in the dark for 48 h at 4 °C. Plants were grown under 120 µmol m^−2^ s^−1^ white light in short day conditions with a 10 h photoperiod, 22/20 °C day/night temperatures, and 70% relative humidity in a Snjider Labs Micro Clima-Series High Specs Plant Growth Chamber.

All genotypes were in a Col-0 background. ABA receptor mutants *q1124* (*pyr1pyl1pyl2pyl4*^13^) and *s112458* (*pyr1pyl1pyl2pyl4pyl5pyl8*^14^) and ABA biosynthesis mutant *nced3/5* have been previously described^13–15^. The *s112458* mutant was kindly provided by Prof Pedro Rodriguez (IBMCP), the *q1124* mutant was kindly provided by Dr Sean Cutler (University of California), the *nced3/5* mutant was kindly provided by Dr Annie Marion-Poll (INRA). Remaining mutants were obtained from the Nottingham Arabidopsis Stock Centre (NASC). *bg1* (SALK_024344/*bg1-3*^40^), *bg2* (SALK_047384/*bg2-3*^18^), *cyp707a1* (SALK_069127/*cyp707a1-1*^41^), *cyp707a3* (SALK_078173^19^). All genotypes were confirmed homozygous populations using PCR. Primers are detailed in Supplementary Table S1. The collection of the plant material used in this study complied with relevant institutional, national, and international guidelines and legislation, and the collection of plant seed was carried out in accordance with national regulations.

### Stomatal aperture bioassays

Experiments were performed on 5 week old plants. Stomatal apertures were measured using leaf discs (4 mm in diameter). For dark-induced closure leaf discs were harvested 2 h after dawn, and incubated in petri dishes containing 10/50 buffer (10 mM MES/KOH, 50 mM KCl, pH 6.2) at 22 °C and illuminated with 120 μmol m^−2^ s^−1^ white light (Crompton Lamps 13 W white) for a further 2 h. Leaf discs were transferred to 10/50 buffer at 22 °C in darkness. Stomatal apertures were measured over a 2 h time course, at 0, 30, 60 and 120 min of dark treatment. A set of control leaf discs were kept in the light for 120 min over the same period (120 min L). For light-induced opening leaf discs were harvested prior to dawn under green light. Leaf discs were incubated in 10/50 buffer at 22 °C in darkness for 2 h before transfer to 120 mmol m^−2^ s^−1^ light. Stomatal apertures were measured over a 2 h time course at 0, 30, 60 and 120 min of light treatment. A set of control leaf discs were kept in the dark for 120 min over the same period (120 min D). For measurement of stomatal aperture leaf discs were imaged using a Leica DMI6000 B inverted microscope and apertures measured using ImageJ (FIJI). For each experimental repeat 30 apertures (over three individual plants) were measured for each treatment, in total over all repeats this amounts to 90 apertures per treatment per genotype. Data was statistically analysed using 2-way ANOVA with Tukey multiple comparison tests.

### Stomatal conductance measurements

Experiments were performed on 6–8 week old plants. A GFS3000 IR gas analyser (Walz) fitted with a 2.5 cm^2^ leaf cuvette was used to measure transpiration. The leaf cuvette was set to 16,000 ppm H_2_O, 400 ppm CO_2_, 22 °C. Flow was set to 750 μmol s^−1^ and impeller speed was set to 7. For darkness applied at midday mature leaves were placed in the cuvette and left to equilibrate for 2 h in a plant growth cabinet under 120 μmol m^−2^ s^−1^ white light. Darkness was imposed for 60/120 min (Figs. 4 and 2 respectively), before reintroduction to 120 μmol m^−2^ s^−1^ white light. For dusk/dawn measurements mature leaves were placed in the cuvette at least 2 h prior to the onset of dusk. Plants were left in the cuvette throughout the night until 2 h post dawn the following day. Stomatal conductance half response times were determined by identifying the time required for transpiration to reach half of the total response over the experiment. Data was analysed using 1-way ANOVAs.

R (version: 4.0.2, url: https://www.r-project.org/) was used to perform statistics and the package ggplot2 used to generate figures^42^.

## Supplementary Information

Supplementary Figures.

Supplementary Table.

## References

[1] Lawson T, Matthews J (2020). Guard cell metabolism and stomatal function. Annu. Rev. Plant Biol..

[2] Jezek M, Blatt MR (2017). The membrane transport system of the guard cell and its integration for stomatal dynamics. Plant Physiol..

[3] Mao J, Zhang Y-C, Sang Y, Li Q-H, Yang H-Q (2005). A role for Arabidopsis cryptochromes and COP1 in the regulation of stomatal opening. Proc. Natl. Acad. Sci..

[4] Costa JM (2015). *OPEN ALL NIGHT LONG*: The dark side of stomatal control. Plant Physiol..

[5] Jiang K (2012). The ARP2/3 complex mediates guard cell actin reorganization and stomatal movement in *Arabidopsis*. Plant Cell.

[6] Isner J-C (2017). Actin filament reorganisation controlled by the SCAR/WAVE complex mediates stomatal response to darkness. New Phytol..

[7] Liang Y-K (2005). AtMYB61, an R2R3-MYB transcription factor controlling stomatal aperture in *Arabidopsis thaliana*. Curr. Biol..

[8] Sierla M (2018). The receptor-like pseudokinase GHR1 is required for stomatal closure. Plant Cell.

[9] Zhang T-Y (2017). Role and interrelationship of MEK1-MPK6 cascade, hydrogen peroxide and nitric oxide in darkness-induced stomatal closure. Plant Sci..

[10] Dittrich M (2019). The role of Arabidopsis ABA receptors from the PYR/PYL/RCAR family in stomatal acclimation and closure signal integration. Nat. Plants.

[11] Merilo E (2013). PYR/RCAR receptors contribute to ozone-, reduced air humidity-, darkness-, and CO_2_-induced stomatal regulation. Plant Physiol..

[12] Leymarie J, Lascève G, Vavasseur A (1998). Interaction of stomatal responses to ABA and CO_2_ in *Arabidopsis**thaliana*. Funct. Plant Biol..

[13] Park S-Y (2009). Abscisic acid inhibits type 2C protein phosphatases via the PYR/PYL family of START proteins. Science.

[14] Gonzalez-Guzman M (2012). Arabidopsis PYR/PYL/RCAR receptors play a major role in quantitative regulation of stomatal aperture and transcriptional response to abscisic acid. Plant Cell.

[15] Frey A (2012). Epoxycarotenoid cleavage by NCED5 fine-tunes ABA accumulation and affects seed dormancy and drought tolerance with other NCED family members: Functional analysis of the NCED5 gene. Plant J..

[16] Iuchi S (2001). Regulation of drought tolerance by gene manipulation of 9-cis-epoxycarotenoid dioxygenase, a key enzyme in abscisic acid biosynthesis in Arabidopsis: Regulation of drought tolerance by AtNCED3. Plant J..

[17] Tan BC, Schwartz SH, Zeevaart JAD, McCarty DR (1997). Genetic control of abscisic acid biosynthesis in maize. Proc. Natl. Acad. Sci..

[18] Xu Z-Y (2012). A vacuolar β-glucosidase homolog that possesses glucose-conjugated abscisic acid hydrolyzing activity plays an important role in osmotic stress responses in Arabidopsis. Plant Cell.

[19] Okamoto M (2009). High humidity induces abscisic acid 8′-hydroxylase in stomata and vasculature to regulate local and systemic abscisic acid responses in arabidopsis. Plant Physiol..

[20] Umezawa T (2006). CYP707A3, a major ABA 8′-hydroxylase involved in dehydration and rehydration response in *Arabidopsis thaliana*. Plant J..

[21] Roelfsema MRG, Hedrich R (2002). Studying guard cells in the intact plant: Modulation of stomatal movement by apoplastic factors. New Phytol..

[22] Chen K (2020). Abscisic acid dynamics, signaling, and functions in plants. J. Integr. Plant Biol..

[23] Yoshida T, Christmann A, Yamaguchi-Shinozaki K, Grill E, Fernie AR (2019). Revisiting the basal role of ABA—Roles outside of stress. Trends Plant Sci..

[24] Tischer SV (2017). Combinatorial interaction network of abscisic acid receptors and coreceptors from *Arabidopsis thaliana*. Proc. Natl. Acad. Sci..

[25] Fujita Y (2009). Three SnRK2 protein kinases are the main positive regulators of abscisic acid signaling in response to water stress in *Arabidopsis*. Plant Cell Physiol..

[26] Miao C (2018). Mutations in a subfamily of abscisic acid receptor genes promote rice growth and productivity. Proc. Natl. Acad. Sci..

[27] Inoue S-I, Kinoshita T (2017). Blue light regulation of stomatal opening and the plasma membrane H^+^-ATPase. Plant Physiol..

[28] Lawson T, Lefebvre S, Baker NR, Morison JIL, Raines CA (2008). Reductions in mesophyll and guard cell photosynthesis impact on the control of stomatal responses to light and CO_2_. J. Exp. Bot..

[29] Tominaga M, Kinoshita T, Shimazaki K (2001). Guard-cell chloroplasts provide ATP required for H+ pumping in the plasma membrane and stomatal opening. Plant Cell Physiol..

[30] Kollist H, Nuhkat M, Roelfsema MRG (2014). Closing gaps: Linking elements that control stomatal movement. New Phytol..

[31] Papanatsiou M (2019). Optogenetic manipulation of stomatal kinetics improves carbon assimilation, water use, and growth. Science.

[32] Lawson T, Vialet-Chabrand S (2019). Speedy stomata, photosynthesis and plant water use efficiency. New Phytol..

[33] Grantz DA, Linscheid BS, Grulke NE (2019). Differential responses of stomatal kinetics and steady-state conductance to abscisic acid in a fern: Comparison with a gymnosperm and an angiosperm. New Phytol..

[34] Mott KA (1988). Do stomata respond to CO_2_ concentrations other than intercellular?. Plant Physiol..

[35] von Caemmerer S (2004). Stomatal conductance does not correlate with photosynthetic capacity in transgenic tobacco with reduced amounts of Rubisco. J. Exp. Bot..

[36] Hsu P-K (2018). Abscisic acid-independent stomatal CO_2_ signal transduction pathway and convergence of CO_2_ and ABA signaling downstream of OST1 kinase. Proc. Natl. Acad. Sci..

[37] Xue S (2011). Central functions of bicarbonate in S-type anion channel activation and OST1 protein kinase in CO_2_ signal transduction in guard cell: CO_2_ signalling in guard cells. EMBO J..

[38] Chater C (2015). Elevated CO_2_-induced responses in stomata require ABA and ABA signaling. Curr. Biol..

[39] Movahedi M (2021). Stomatal responses to carbon dioxide in light require abscisic acid catabolism in Arabidopsis. Interface Focus.

[40] Ondzighi-Assoume CA, Chakraborty S, Harris JM (2016). Environmental nitrate stimulates abscisic acid accumulation in Arabidopsis root tips by releasing it from inactive stores. Plant Cell.

[41] Okamoto M (2006). CYP707A1 and CYP707A2, which encode abscisic acid 8′-hydroxylases, are indispensable for proper control of seed dormancy and germination in arabidopsis. Plant Physiol..

[42] Wickham H (2009). ggplot2.

