# Supplementary material for: ABA signalling and metabolism are not essential for dark-induced stomatal closure but affect response speed
Source: Sci Rep. 2021 Mar 11;11:5751. doi: 10.1038/s41598-021-84911-5 (PMC7952387; doi:10.1038/s41598-021-84911-5)
Supplement: Supplementary file 1 — Supplementary Figures. [file 41598_2021_84911_MOESM1_ESM.pdf]

ABA signalling and metabolism are not essential for dark-induced stomatal closure but affect response speed

Pridgeon, Ashley J. and Hetherington, Alistair M. *

School of Biological Sciences, Life Sciences Building, University of Bristol, 24 Tyndall Avenue, Bristol, BS8 1TQ

* Correspondence to Alistair.Hetherington@bristol.ac.uk

Supplementary Figure Legends

Supplementary Figure 1 Absolute change in stomatal aperture of ABA signalling, biosynthesis and degradation mutants to darkness

The absolute change in leaf disc stomatal aperture relative to time 0 for **a)** ABA receptor mutants (*q1124* and *s112458*), **b)** *nced3/5*, **c)** ABA activation mutants (*bg1* and *bg2*) and **d)** ABA degradation mutants (*cyp707a1* and *cyp707a3*) over a 120 min time course in response to darkness (absolute apertures are presented in Fig. 1). 120 mins L represents leaf discs left in light over the 120 min time course. n = 90 from 9 individual plants over 3 independent experiments. Data is presented in boxplots showing the median and interquartile range of each group. The upper and lower whiskers represent data within 1.5 * the interquartile range. All data values are represented by points. Data statistically analysed using 2-way ANOVA with Tukey multiple comparison tests, letters denote significant differences at $p < 0.05$.

Supplementary Figures

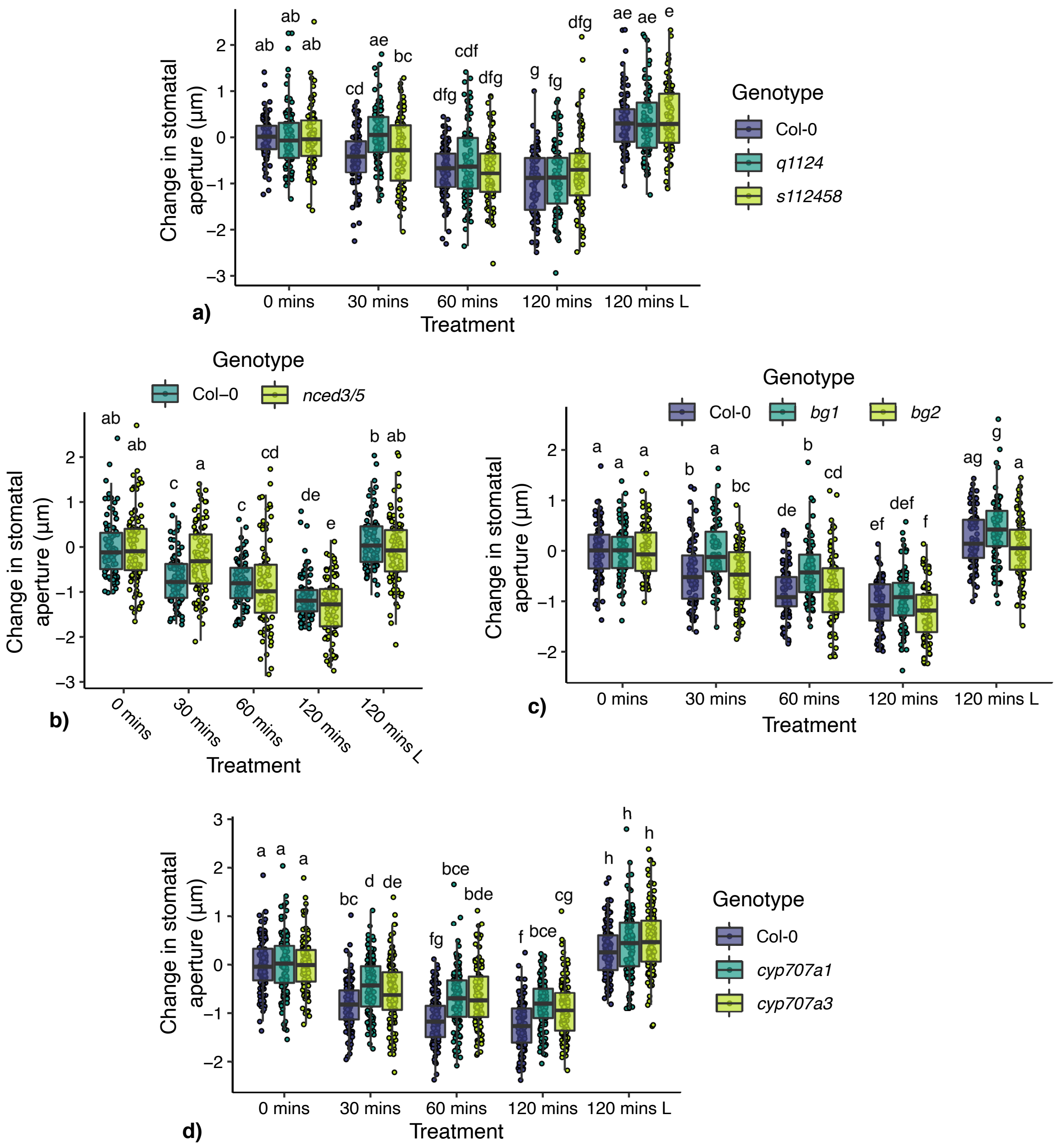

Supplementary Figure 1 Absolute change in stomatal aperture of ABA signalling, biosynthesis and degradation mutants to darkness

The absolute change in leaf disc stomatal aperture relative to time 0 for **a)** ABA receptor mutants (*q1124* and *s112458*), **b)** *nced3/5*, **c)** ABA activation mutants (*bg1* and *bg2*) and **d)** ABA degradation mutants (*cyp707a1* and *cyp707a3*) over a 120 min time course in response to darkness (absolute apertures are presented in Fig. 1). 120 mins L represents leaf discs left in light over the 120 min time course. n = 90 from 9 individual plants over 3 independent experiments. Data is presented in boxplots showing the median and interquartile range of each group. The upper and lower whiskers represent data within 1.5 * the interquartile range. All data values are represented by points. Data statistically analysed using 2-way ANOVA with Tukey multiple comparison tests, letters denote significant differences at $p < 0.05$.
